# Long-term Nephrotoxicity after PRRT: Myth or Reality

**DOI:** 10.7150/thno.92487

**Published:** 2024-01-01

**Authors:** Richard P. Baum, Xin Fan, Vivianne Jakobsson, Fei Yu, Christiane Schuchardt, Xiaoyuan Chen, Jingjing Zhang

**Affiliations:** 1 CURANOSTICUM Wiesbaden-Frankfurt, Center for Advanced Radiomolecular Precision Oncology, Wiesbaden, Germany.; 2 Theranostics Center for Molecular Radiotherapy and Precision Oncology, ENETS Center of Excellence, Zentralklinik Bad Berka, Bad Berka, Germany.; 3 Department of Diagnostic Radiology, Yong Loo Lin School of Medicine, National University of Singapore, Singapore, Singapore.; 4 Clinical Imaging Research Centre, Centre for Translational Medicine, Yong Loo Lin School of Medicine, National University of Singapore, Singapore, Singapore.; 5 Department of Nuclear Medicine, Shanghai Tenth People's Hospital, Tongji University School of Medicine, Shanghai, China.; 6 Institute of Nuclear Medicine, Tongji University School of Medicine, Shanghai, China.; 7 Nanomedicine Translational Research Program, NUS Center for Nanomedicine, Yong Loo Lin School of Medicine, National University of Singapore, Singapore, Singapore.; 8 Department of Surgery, Chemical and Biomolecular Engineering, and Biomedical Engineering, Yong Loo Lin School of Medicine and College of Design and Engineering, National University of Singapore, Singapore, Singapore.; 9 Institute of Molecular and Cell Biology, Agency for Science, Technology, and Research (A*STAR), 61 Biopolis Drive, Proteos, Singapore, Singapore.

**Keywords:** peptide receptor radionuclide therapy (PRRT), nephrotoxicity, long-term, lutetium-177 (^177^Lu), yttrium-90 (^90^Y), somatostatin analogs

## Abstract

**Rationale:** The kidneys are commonly considered as the potential dose-limiting organ for peptide receptor radionuclide therapy (PRRT), making the risk of nephrotoxicity a primary concern. This retrospective analysis with prospective documentation and long-term follow-up aims to assess the risk of nephrotoxicity after PRRT in a large cohort of patients with neuroendocrine neoplasms (NENs) treated at our institution over the past 18 years.

**Methods:** A total of 1361 NEN patients treated with 1-10 cycles of ^177^Lu-DOTA-TOC/-NOC/-TATE, ^90^Y-DOTA-TOC/-NOC/-TATE, DUO-PRRT (sequential administration of ^90^Y- and ^177^Lu-), or TANDEM-PRRT (combination of ^90^Y- and ^177^Lu- on the same day concomitantly) were included in this analysis. All parameters were prospectively documented in a structured database comprising over 250 items per patient and retrospectively analyzed. Kidney function, including serum creatinine, blood urea nitrogen, cGFR, and electrolytes, was evaluated before each PRRT cycle and during follow-up. Restaging was regularly performed at 6-month intervals until death. Treatment-related adverse events were graded according to the Common Terminology Criteria for Adverse Events (CTCAE v.5.0).

**Results:** Between 2000 and 2018, a total of 5409 cycles of PRRT were administered to 1361 NEN patients. Follow-up after complete treatment was available for 1281 patients receiving 4709 cycles of PRRT, with a median follow-up time of 69.2 months (interquartile range, 32.8-110.5 months) and a maximum follow-up time of 175 months. Baseline creatinine levels were normal in 1039/1281 (81.1%) subjects, while grade 1 (G1) renal insufficiency was present in 221/1281 (17.3%) prior to PRRT. G2 was present in 19/1281 (1.5%), and G3 in 2/1281 (0.2%). After treatment, the proportion of G3/G4 grade patients only increased from 0.2% to 0.7%. Mean creatinine levels increased from a baseline of 0.90 ± 0.30 to 1.01 ± 0.57 mg/L (80.0 ± 26.7 to 89.4 ± 50.8 μmol/L) after treatment. In our main analysis cohort of 1244 patients (4576 cycles), 200 patients experienced an increase in CTCAE creatinine grade. Age, number of treatment cycles, type of radionuclides, and length of follow-up time were the main factors affecting CTCAE creatinine grading after treatment. When comparing the subgroups treated with different radionuclides, the risk of nephrotoxicity after ^90^Y treatment alone and the ^90^Y/^177^Lu combination group was higher than after ^177^Lu treatment alone. In the ^90^Y treatment subgroup, the two significant risk factors for an increased CTCAE creatinine grade were identified to be age (≥60) and a long follow-up time.

**Conclusions**: This retrospective analysis with prospective documentation in a large cohort of 1281 NEN patients receiving 4709 cycles of PRRT co-administered with renal protection, treated through the individualized approach at a single institution over 18 years, did not reveal any evidence of long-term PRRT-related renal toxicity. The results of our study suggest that with the use of proper renal protection, nephrotoxicity due to PRRT is more likely a myth than a reality.

## Introduction

Peptide receptor radionuclide therapy (PRRT) using radiolabeled somatostatin analogs has shown significant success in managing neuroendocrine neoplasms (NENs) and has become an established treatment for patients with unresectable or metastatic, progressive, well-differentiated neuroendocrine tumors (NETs) 1. PRRT offers benefits in progression-free survival (PFS) and overall survival (OS) for metastasized and progressive NENs, regardless of prior therapies. Lutathera (^177^Lu-DOTATATE) has received approval from the European Medicines Agency (EMA) and the U.S. Food and Drug Administration (FDA) for treating metastatic, progressive, well-differentiated (G1/G2), somatostatin receptor-positive gastroenteropancreatic neuroendocrine tumors (GEP-NETs) in adults 2, 3. While PRRT has demonstrated impressive results, concerns about long-lasting side effects, such as nephrotoxicity, have been raised and remain a major limitation in individual patient treatment plans. Ensuring safety and exercising caution in implementing relatively new treatments is of utmost importance, and the lack of long-term follow-up data is a significant reason for clinician hesitancy.

During PRRT, the kidneys are generally considered as the organs with the potential for dose-limitation, making renal toxicity a primary concern 4. Renal irradiation primarily occurs because radiolabeled peptides are filtered through the glomerular capillaries in the kidneys and efficiently reabsorbed by cells in the proximal tubule of the nephron, where a significant amount of radioactivity is retained 5, 6. Additionally, somatostatin receptor subtype-2 (SSTR2) expression in human kidneys, including vasa recta, tubular cells of the cortex, and distal tubule cells, also contributes to the total renal uptake of radiolabeled somatostatin analogs 7.

Currently, kidneys are protected during PRRT by co-infusing competitive inhibitors of reabsorption that interfere with the interaction between radiolabeled peptides and renal endocytic receptors. Nephrotoxicity may also be reduced by dose fractionation, using radioprotectors, or employing mitigating agents. The maximum tolerable absorbed dose from PRRT of kidneys has previously been extrapolated from external-beam radiation therapy (EBRT) and established to be 23 Gy 8-10. Data from EBRT suggest that total doses associated with a 5% and 50% risk of renal failure at 5 years are 18-23 Gy and 28 Gy, respectively, with fractions of 0.5-1.25 Gy. However, it remains uncertain whether these results can be directly applied to PRRT with radiolabeled somatostatin analogs due to the sustained but lower radiation dose rate and the different physical properties of the administered radioactive particles (mainly beta and alpha emitters), and it is crucial to observe and document any nephrotoxicity or clinically significant loss of renal function over an extended period after PRRT, especially in patients who undergo multiple PRRT cycles and are exposed to a longer total duration of irradiation 4.

Given the limited long-term data on nephrotoxicity related to PRRT, this retrospective analysis with prospective documentation and long-term follow-up aims to assess the nephrotoxicity of PRRT in a large cohort of NEN patients (1361) treated at our institution over the past 18 years.

## Materials and Methods

### Patients

The study was conducted in compliance with the legal requirements, including ethical guidelines and local radiation protection regulations. The research was carried out following the approved guidelines of the local ethical committee at Zentralklinik Bad Berka and in accordance with German regulations published by the Federal Office for Radiation Protection, ensuring radiation safety. All patients included in the study had progressive neuroendocrine neoplasms (NENs) and had exhausted conventional therapeutic options. Written informed consent was obtained from all patients for the treatment and the use of their anonymized clinical data for scientific purposes. Age was calculated based on the time of the first PRRT treatment. The study included adult patients with histopathologically confirmed NENs, primarily characterized by high SSTR expression in the majority of lesions. Patients who received peptide receptor chemo-radionuclide therapy (PRCRT), which combines PRRT with chemotherapy, during PRRT or at restaging, except for TACE, were excluded from this study.

### Radiopharmaceutical Preparation

The 1,4,7,10-tetraazacyclododecane-N,N',N'',N'''-tetraacetic acid (DOTA)-conjugated somatostatin analogs, namely DOTATOC, DOTATATE, and DOTANOC, were labeled with ^68^Ga, ^177^Lu, and ^90^Y, respectively^11^, at our institutional radiopharmacy following a strict Good Manufacturing Practice (GMP) protocol. The radionuclide ^68^Ga was obtained in-house from a ^68^Ge/^68^Ga generator. Our hospital has developed a highly efficient procedure for NaCl-based ^68^Ga labeling 12. ^177^Lu and ^90^Y were sourced from different manufacturers. The labeling of DOTA-conjugated peptides with ^177^Lu and ^90^Y was performed following previously published methods 13. Quality control was conducted using high-performance liquid chromatography (HPLC), ensuring that the radiochemical purity consistently exceeded 99%.

### Renal protection

For renal protection, each patient received a co-infusion of a reno-protective amino acid mixture (1,600 mL of 5% lysine HCl and 10% L-arginine HCl, with a pH of 7.4 and an osmolarity of 400 mOsm/L) 14, 15. The infusion was initiated at least 30 min before the administration of the radiotherapeutics and continued for 4 h thereafter. The radiopharmaceutical was administered over a period of 10-15 min using a second infusion pump system. In patients with impaired renal function (glomerular filtration rate, <60 mL/min) and for the application of ^90^Y, 4% Gelofusine (B. Braun Melsungen AG) was infused according to patients' weights for additional nephroprotection. Starting from January 2007, patients treated with ^90^Y/^177^Lu-DOTATATE also received a co-infusion of succinylated gelatin 4, 16. The protocol for this co-infusion involved initially administering 1 mL/kg (body weight) of 4% gelafusal (500 mL containing 20 g of gelatin) as a bolus over 10 min before the start of the therapy. Following the radiopeptide infusion, the gelofusal infusion was initiated at a rate of 0.02 mL/kg (body weight) per minute over a period of 3 h. Blood pressure and heart rate were continuously monitored throughout the therapy. Each patient was adequately hydrated, ensuring an intake of at least 1 L of mineral water before the therapy and 2-3 L thereafter. Special care was taken not to induce hypervolemia or hyperosmolarity in patients with carcinoid heart disease and high-grade tricuspid regurgitation. In cases where there was evidence of renal obstruction, either physiologic or pathological, an intravenous injection of 20-40 mg of furosemide in 1.5-2 L of deltajonin was administered over a period of 2-4 h after the therapy.

### Treatment regimen

Patients received radionuclide therapy in different subsets, including ^177^Lu-PRRT, ^90^Y-PRRT, DUO-PRRT (sequential administration of ^90^Y- and ^177^Lu-PRRT, involving highly individualized and interdisciplinary care), and TANDEM-PRRT (combination of ^90^Y- and ^177^Lu-PRRT administered on the same day concomitantly). The administered activity was determined individually based on the Bad Berka Score (BBS) 17. The BBS takes into account factors such as the uptake of tumor lesions observed through ^68^Ga-SSTR PET/CT (performed before each treatment cycle), renal function, hematological reserve, liver involvement, extra-hepatic tumor burden, Ki-67 index, tumor grade, ^18^F-FDG PET/CT status, tumor dynamics (doubling time, new lesions), weight loss, time since the initial diagnosis, functional activity of the tumor, previous treatments, and the general status of the patient (assessed using the Karnofsky Performance Scale). The decision to use ^90^Y and/or ^177^Lu was based on considerations such as tumor mass, renal and hematological function, previous therapies (particularly chemotherapy), SUV, and other factors outlined in the BBS.

### Renal function assessment

All parameters were prospectively documented in a structured database, which included over 250 items per patient, and subsequently analyzed retrospectively. Kidney function, including serum creatinine, blood urea nitrogen, cGFR (glomerular filtration rate), and electrolyte levels, was evaluated before each PRRT cycle and during follow-up. Regular restaging assessments were conducted at 6-month intervals until the time of death. Treatment-related adverse events were graded according to the Common Terminology Criteria for Adverse Events (CTCAE v.5.0). The CTCAE classification categorizes adverse events on a scale of 1 to 5, with grade 3 and higher indicating a serious event.

### Follow-up

Restaging with SSTR PET/CT was conducted every 3-4 months following PRRT. If the disease remained stable or showed remission (complete or partial), restaging with SSTR PET/CT was performed every six months until disease progression was observed on imaging. SSTR and FDG PET/CT scans were conducted using a Siemens Biograph Duo until January 2014, and subsequently with the Biograph mCT Flow 64 from Siemens Medical Solutions AG, Erlangen, Germany. Contrast-enhanced CT (using the Biograph mCT Flow 64) was obtained after intravenous administration of 60-100 mL of nonionic iodinated contrast. The acquired images were evaluated by two experienced nuclear medicine specialists. PRRT was resumed if disease progression occurred after a therapy interval of more than six months, referred to as the next "treatment phase" of PRRT.

### Statistical analysis

Continuous variables were presented as mean ± standard deviation. The Wilcoxon signed-rank test was utilized to compare creatinine values before and after treatment. Binary logistic regression analysis was performed to identify factors influencing creatinine levels, considering variables identified through single factor analysis. A multivariate binary logistic regression analysis was conducted to determine the final potential factors affecting decreased renal function. Patients were divided into groups with or without renal function impairment based on whether the CTCAE classification increased. Furthermore, patients were categorized into two groups based on whether renal function was impaired prior to treatment or only after treatment. All statistical tests were two-tailed, and P values less than 0.05 were considered statistically significant.

## Results

A total of 1361 patients were enrolled in this study, and ultimately, 1281 patients with comprehensive pre- and post-treatment renal function follow-up data were included in the analysis. Table 1 provides an overview of the baseline characteristics of the 1281 included patients with NENs.

### Change in nephrotoxicity grading before and after treatment

The study involved a total of 1361 patients who underwent 5409 cycles of PRRT using ^177^Lu, ^90^Y, or a combination of both (Table S1), between the years 2000 and 2018. Follow-up data after complete treatment were available for 1281 patients, accounting for 4709 cycles. The median follow-up time was 69.2 months (IQR, 32.8 to 110.5 months), with a maximum follow-up time of 175 months.

At baseline, 81.1% (1039/1281) of the subjects had normal creatinine levels, while 17.3% (221/1281) had grade 1 (G1) renal insufficiency (RI), 1.5% (19/1281) had G2 RI, and 0.2% (2/1281) had G3 RI. Following treatment, the baseline creatinine level increased from 0.90 ± 0.30 to 1.01 ± 0.57 mg/L (80.0 ± 26.7 to 89.4 ± 50.8 μmol/L). In the follow-up study cohort, the proportion of patients with CTCAE creatinine G3/G4 classification only rose from 0.2% to 0.7% after treatment.

### Effects of the number of treatment cycles on renal function

**Figure 1:**
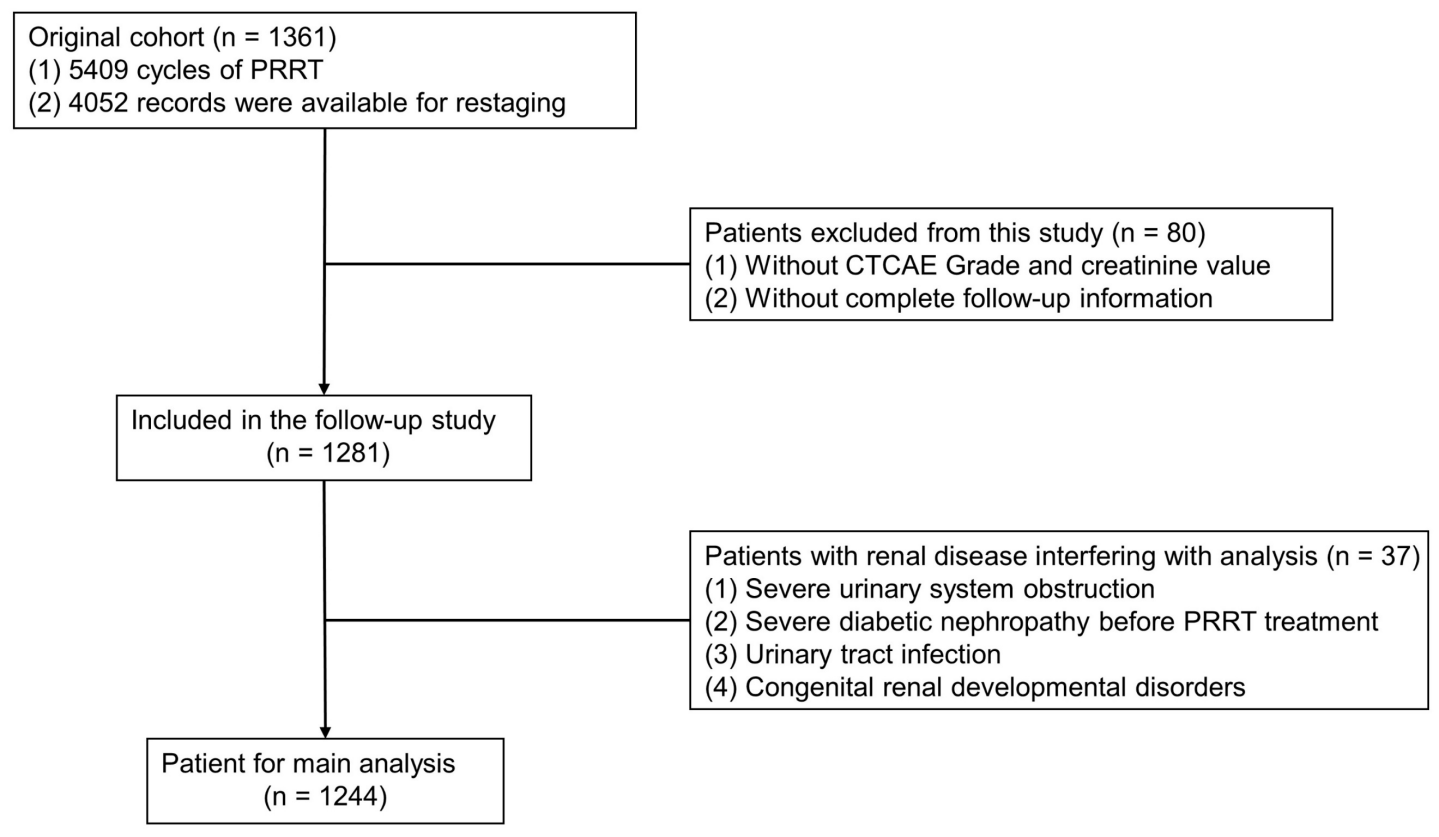
Flow chart of the patient screening process in the original cohort.

**Figure 2:**
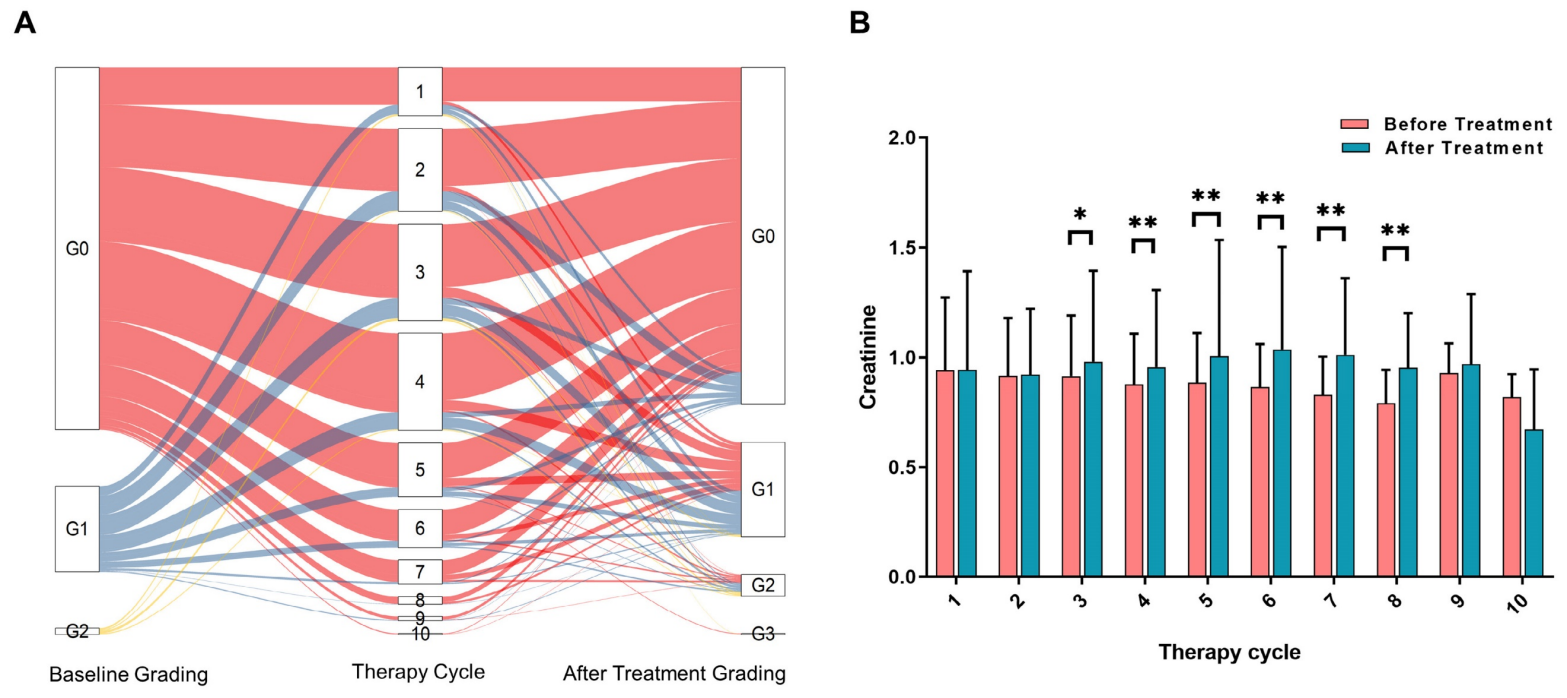
** A.** Changes in CTCAE-based nephrotoxicity assessment using creatinine before and after different treatment cycles (all subsets). The color red represents patients starting with a G0 classification, blue represents patients starting with a G1 classification, and yellow represents patients starting with a G2 classification. B. Histogram showing the distribution of creatinine levels before and after each radionuclide therapy cycle (all subsets). The significance levels are denoted as * P < 0.05, ** P < 0.01, and *** P < 0.001.

To accurately assess the risk factors affecting renal function, we excluded patients who had renal function damage resulting from other causes such as severe urinary system obstruction, pre-existing renal insufficiency, diabetic nephropathy, congenital renal developmental disorders, and urinary tract infection. Ultimately, a total of 1244 patients were included in the analysis of risk factors (Figure 1). Among these patients, a total of 4576 treatment cycles were performed, and 620 patients underwent more than 3 cycles of PRRT. The changes in creatinine grading based on CTCAE before and after different treatments are depicted in Figure 2A. Analysis of serum creatinine levels revealed a significant increase from the 4^th^ treatment cycle (P < 0.01) (Figure 2B). Two clinical cases in which renal function did not deteriorate after long-term follow-up within the first 4 treatment cycles are shown in Figures S1 and S2.

### Renal function in relation to the specific radionuclide used for treatment

**Figure 3:**
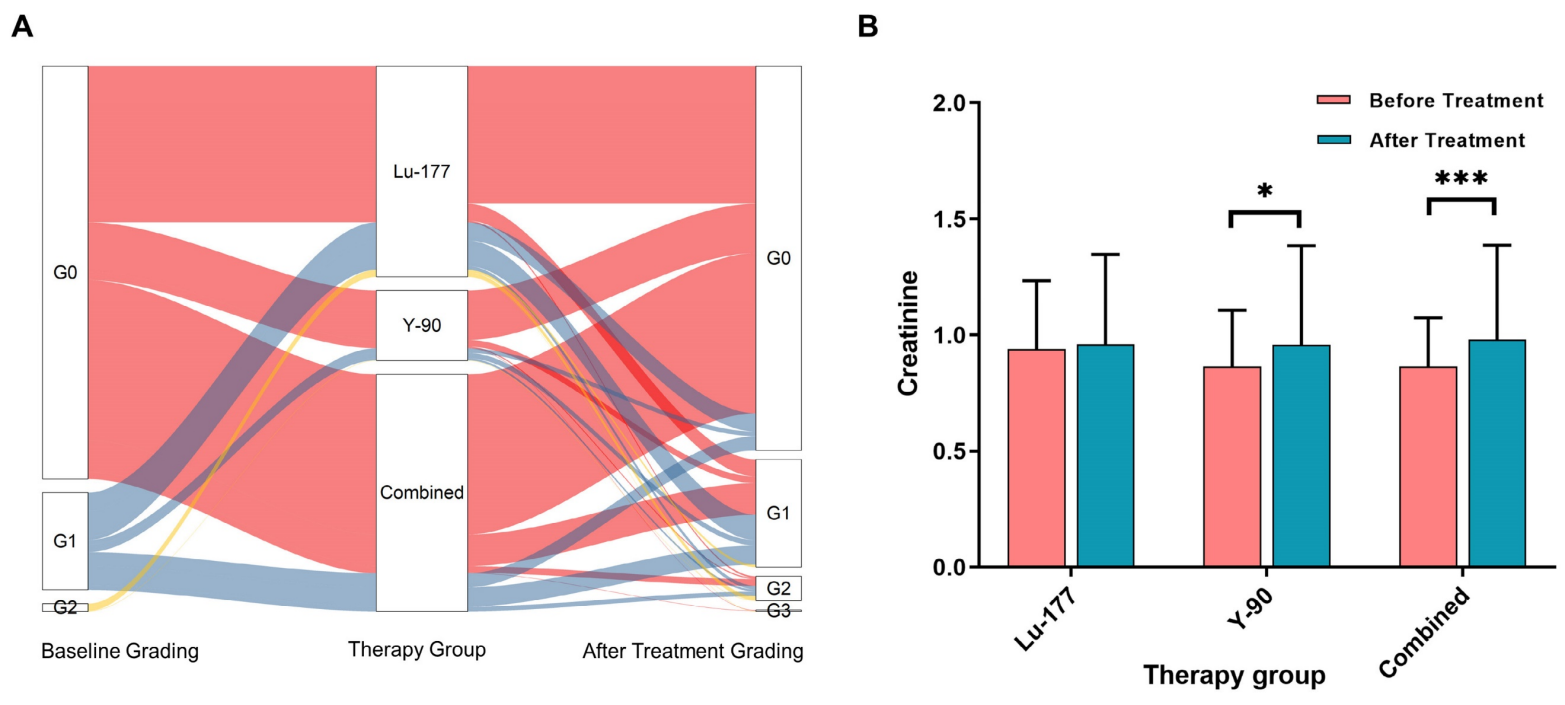
** A.** shows the changes in creatinine grade before and after different treatments. **B.** presents the creatinine levels before and after treatment in the following groups: ^177^Lu alone, ^90^Y alone, and combined treatment (including sequential or simultaneous treatment with both nuclides).

Figure 3A displays the creatinine grades before and at different times after treatment. In the ^177^Lu treatment group, no statistically significant difference was observed in creatinine levels before and after treatment (P = 0.431). However, in the treatment group with ^90^Y (including ^90^Y alone and combined treatment with ^177^Lu and ^90^Y), significant differences were observed in creatinine levels (P = 0.014 and < 0.001), as depicted in Figure 3B.

### Univariate and Multivariate Analysis for Progression of Renal Impairment

In our cohort of 1244 patients, 200 individuals (16.1%) experienced an increase in creatinine levels based on CTCAE grading. Among them, 167 patients (13.4%) had an increase of 1 grade, 31 patients (2.5%) had an increase of 2 grades, and 2 patients (0.2%) had an increase of 3 grades. Of the 2 patients with a three-grade increase in creatinine, 1 received 5 cycles of ^90^Y PRRT (4, 3.5, 4.6, 5, 3.5 GBq or 108, 94.5, 123, 135, 94.5 mCi), while the other received 2 cycles of ^90^Y (2.8, 5 GBq or 75.6,135 mCi) and 3 cycles of ^177^Lu (6, 7.6, 5 GBq or 162, 205.2, 135 mCi) PRRT.

The cohort included three treatment subgroups: 506 patients (40.7%) in the ^177^Lu alone treatment group, 169 patients (13.6%) in the ^90^Y alone treatment group, and 569 patients (45.7%) in the combination treatment group. Treatment dose information for the three subgroups is shown in Table 3.

**Figure 4:**
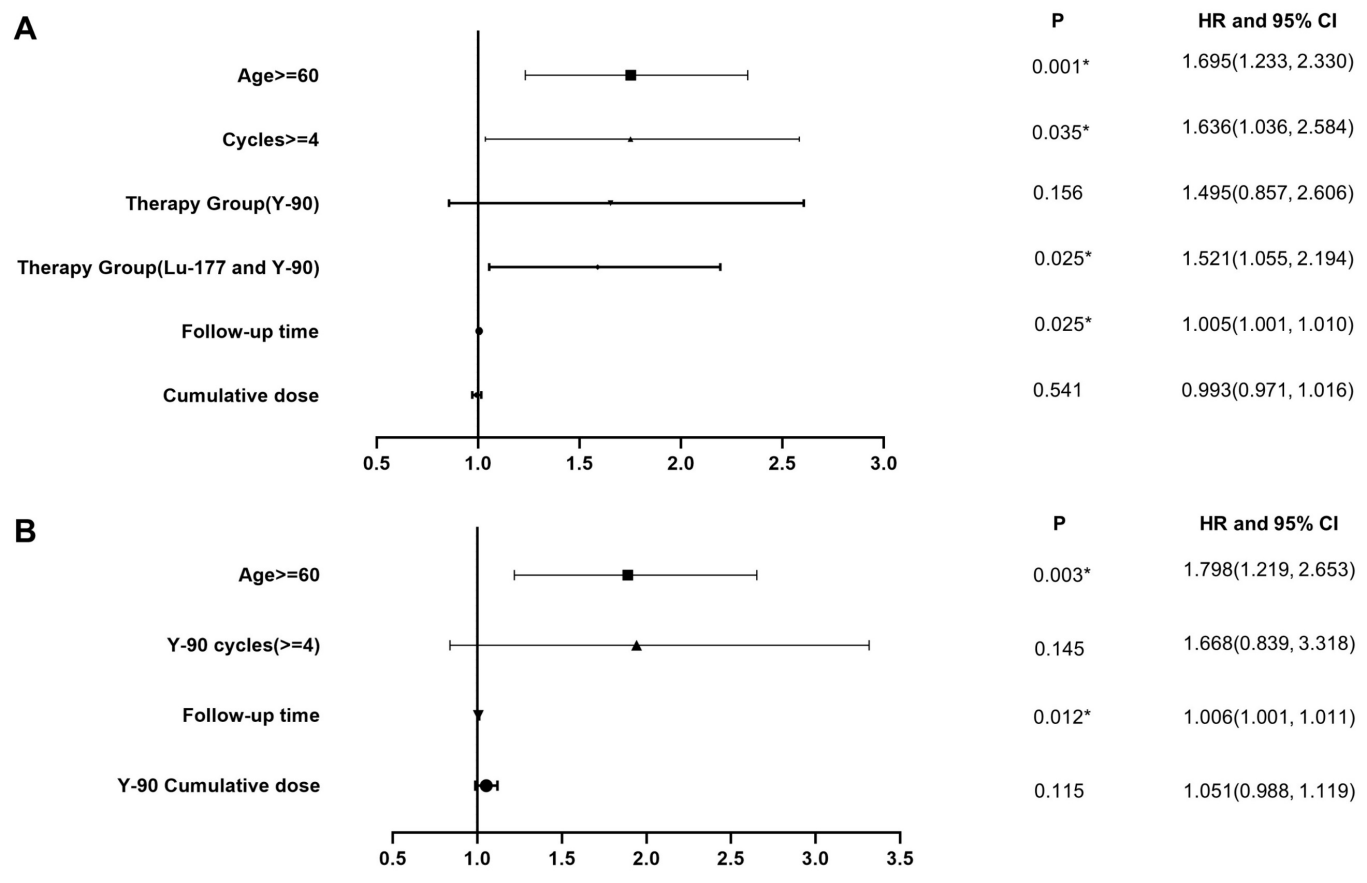
**A.** Multivariate analysis in the overall study cohort. **B.** Multivariate analysis of the subgroup which received PRRT with ^90^Y labeled somatostatin analogs.

We conducted the main analysis in 1244 patients and found that age, follow-up time, number of treatment cycles, and choice of radionuclides were factors contributing to decreased renal function, as indicated by an increased CTCAE creatinine grade (Figure 4A, Table 4). The risk of decreased renal function after ^90^Y PRRT alone and combined treatment was higher than that after ^177^Lu PRRT alone. Subsequently, based on the previous differential analysis, we performed subgroup analyses in the ^90^Y group. The significant risk factors for renal function impairment in the ^90^Y PRRT subgroup were age older than 60 and a long follow-up time (Figure 4B, Table 5). Serial renal function assessments in a patient treated with 10 PRRT cycles over 9 years, measured by the tubular extraction rate (TER) using ^99m^Tc-MAG3, showed no worsening of kidney function (Figure S3).

## Discussion

To our knowledge, this study represents the largest cohort of patients with NENs and the longest follow-up time to date, including various approaches to PRRT. In this retrospective analysis, we included renal function data from multiple treatments, different treatment doses, and long-term follow-up. We evaluated changes in renal function before and after treatment in a total of 1281 patients, encompassing 4709 treatment cycles, with a median follow-up time of 69.2 months and a maximum follow-up time of 175 months. Our results indicate that PRRT is a safe treatment with a very low incidence of severe nephrotoxicity. This conclusion is further supported by specific cases. One patient showed no significant change in tubular extraction rate (TER) during a follow-up period of over eight years after three cycles of PRRT (Figure S3). Even in a patient with grade 2 renal impairment before treatment, renal function did not worsen after 3 cycles of PRRT (Figure S2).

In the follow-up study cohort, the proportion of patients with CTCAE grade 3 or 4 creatinine levels increased from 0.2% to 0.7% after treatment. In the subsequent main analysis cohort, we excluded 37 patients with concurrent diseases that could have affected the analysis results. Among the three patients who developed grade 4 creatinine increase after treatment, 1 had severe renal insufficiency (grade 3) before treatment, 1 had recurrent urinary tract infections, and 1 had severe kidney stones with colic.

In the results of the univariate analysis, we identified differences in creatinine levels before and after different treatment cycles and types of radionuclides as initial screening variables to set cutoff values for further analysis. Through multivariate analysis, we included as complete and relevant clinical information as possible to determine the degree of influence of different factors on renal function impairment. The results revealed that the main risk factors associated with increased CTCAE grade were patient age, length of follow-up time, number of treatment cycles, and types of radionuclides. In the subgroup analysis of patients treated with ^90^Y PRRT, age and follow-up time were significant risk factors, with no significant difference between the ^90^Y group and the ^90^Y/^177^Lu combination group. These findings support the lack of nephrotoxicity after PRRT, particularly with ^177^Lu.

Our findings are consistent with previous studies. Kunikowska *et al.* reported a slightly higher but acceptable risk of nephrotoxicity with ^90^Y in their long-term follow-up of 53 Caucasian patients with metastatic NENs 18. Bodei *et al.* conducted a retrospective analysis and found that nephrotoxicity of any grade, transient or persistent, occurred in 34.6% of patients after PRRT with ^90^Y and ^177^Lu, with severe nephrotoxicity observed in 1.5% of patients. They also reported a higher proportion of severe nephrotoxicity in the ^90^Y and ^90^Y + ^177^Lu treatment groups compared to the ^177^Lu alone group 19. These results were further supported by other studies, including those by Rolleman *et al.* and Valkema et al., which highlighted the differences in renal toxicity between ^90^Y and ^177^Lu 20, 21. In the NETTER-1 trial, no evidence of renal toxicity was observed in the ^177^Lu-DOTATATE group during the observed time frame 3. Studies conducted by the Basel group and the Milan group also demonstrated the importance of nephroprotection in minimizing renal damage during PRRT 22-28.

In this study, we did not include dose-related risk factors in the overall population due to the correlation between the number of treatments and treatment dose, which could have resulted in biased outcomes. However, the overall results indicate no significant impairment of renal function, which aligns with the findings of previous studies 29. Importantly, our study provides long-term follow-up results to support our conclusions, and benefitted from a large cohort size, a comprehensive documentation process, and the use of optimal nephroprotection protocols. These factors contribute to the robustness and reliability of our findings.

However, it is important to acknowledge the limitations of our study, such as its retrospective nature and the reliance on data from a single institution. Future prospective studies, preferably involving multiple centers, could consider including normal populations simultaneously to account for the effects of age and time on renal function. Additionally, the absence of glomerular filtration rate (GFR) and other renal function imaging data in the entire population led us to rely on serum creatinine as an evaluation tool, which is a quantitative measure of renal function. Another limitation is the lack of dosimetry data to assess actual organ uptake, which could have further strengthened the observed correlation between function decline and radiation dose delivered. However, previous studies did not find a significant correlation between dose delivered and the severity of nephrotoxicity. Therefore, we cautiously hypothesize that the inclusion of dosimetry data would not significantly alter the results and the conclusions of this study while acknowledging that dosimetry remains crucial for evaluating novel radiopharmaceuticals.

## Conclusion

The efficacy of PRRT has been well established, but concerns regarding its long-term safety, specifically the risk of nephrotoxicity, have persisted. In this study, we conducted a retrospective analysis with prospective documentation in a large cohort of 1281 patients with neuroendocrine neoplasms (NENs). These patients underwent a total of 4709 cycles of PRRT with optimal nephroprotection over 18 years at a single institution. Our findings indicate that there is no significant evidence of long-term nephrotoxicity associated with PRRT.

Based on the results of our study, concerns about PRRT-induced nephrotoxicity may be unfounded. The absence of significant nephrotoxicity in our cohort suggests that the notion of PRRT-related nephrotoxicity may be more of a myth than a reality. This is an important finding, as it provides reassurance regarding the safety of PRRT as a treatment option for patients with tumors expressing SSTR.

It is worth noting that our study benefitted from a large cohort size, a comprehensive documentation process, and the use of optimal nephroprotection protocols. These factors contribute to the robustness and reliability of our findings. However, it is essential to acknowledge the limitations of our study, such as its retrospective nature and the reliance on data from a single institution. Further studies, including prospective investigations involving multiple centers, will be valuable in confirming and expanding upon our results.

In conclusion, our study provides evidence that PRRT is a safe treatment option for patients with SSTR-expressing tumors. The absence of significant long-term nephrotoxicity supports the use of PRRT as an effective and well-tolerated therapeutic approach for NENs.

## Supplementary Material

Supplementary figures and table.

## Figures and Tables

**Table 1:** Patients' characteristics in the 1281 patients

Characters	Number (n)	Percent (%)
Gender		
Male	718	56.0
Female	563	44.0
Age (years)	59.57 ± 11.44	
Other disease		
Yes	1227	95.8
No	54	4.2
Primary site		
Midgut	411	32.1
Pancreas	420	32.8
Stomach	14	1.1
Cup	156	12.2
Thymus/Mediastinum	17	1.3
Lung	82	6.4
Colon	4	0.3
Rectum	55	4.3
Others	122	9.5
Therapy cycle		
1	135	10.5
2	238	18.6
3	268	20.9
4	275	21.5
5	154	12.0
6	107	8.4
7	68	5.3
8	21	1.6
9	12	0.9
10	3	0.2
Treatment Regimen		
^177^Lu-PRRT	526	41.1
^90^Y-PRRT	172	13.4
DUO-PRRT	556	43.4
TANDEM-PRRT	27	2.1

**Table 2:** Changes in Renal Function Classification and Creatinine Before and After Treatment in 1281 patients (All Subsets)

	Before Treatment		After Treatment		
	Number (n)	Percent (%)	Number (n)	Percent (%)	*P*
**Creatinine Grading**					<0.001
G0	1039	81.1	923	72.1	
G1	221	17.3	272	21.2	
G2	19	1.5	77	6.0	
G3	2	0.2	6	0.5	
G4	0	0	3	0.2	
**Creatinine**	0.90 ± 0.30		1.01 ± 0.57		<0.001

Note: The P-value indicates the statistical significance of the change in renal function classification and creatinine levels before and after treatment.

**Table 3:** The treatment dose information about the different subgroup

	^177^Lu (n=506)	^90^Y (n=169)	Combination (n=569)
Cumulative ^177^Lu dose	19.9 ± 10.1 GBq (537.3 ± 272.7 mCi) (1.5 - 64.1 GBq)	\	17.6 ± 10.1 GBq (475.2 ± 272.7 mCi) (1.7 - 60.9 GBq)
Cumulative ^90^Y dose	\	7.6 ± 4.4 GBq (205.2 ± 118.8 mCi) (1.5 - 20.6 GBq)	6.5 ± 3.8 GBq (175.5 ± 102.6 mCi) (1.5 - 23.6 GBq)
Maximum ^177^Lu dose	7.0 ± 1.2 GBq (189 ± 32.4 mCi) (1.5 - 10.8 GBq)	\	7.0 ± 1.2 GBq (189 ± 32.4 mCi) (1.7 - 12.0 GBq)
Maximum ^90^Y dose	\	3.9 ± 1.1 GBq (105.3 ± 29.7 mCi) (1.5 - 7.0 GBq)	6.5 ± 3.8 GBq (175.5 ± 102.6 mCi) (1.0 - 9.5 GBq)
Treatment cycles	1-9	1-7	2-10

**Table 4:** Univariate and Multivariate Analyses of the Main Analysis Cohort

	Univariate analysis		Multivariate analysis	
Factors	HR and 95% CI	*P*	HR and 95% CI	*P*
**Age**				
<60				
≥60	1.575(1.152, 2.152)	0.004*	1.695(1.233, 2.330)	0.001*
**Gender**				
Male				
Female	1.341(0.990, 1.816)	0.058		
**Therapy cycle**				
<4 cycles				
≥4 cycles	1.848(1.354, 2.523)	<0.001*	1.636(1.036, 2.584)	0.035*
**Therapy group**				
^177^Lu				
^90^Y	1.413(0.864, 2.311)	0.168	1.495(0.857, 2.606)	0.156
Combined	1.842(1.312, 2.586)	<0.001*	1.521(1.055, 2.194)	0.025*
**Follow-up time**	1.007(1.003, 1.011)	0.001*	1.005(1.001, 1.010)	0.025*
**Cumulative dose**	1.016(1.002, 1.029)	0.022*	0.993(0.971, 1.016)	0.541

**Table 5:** Univariate and multivariate analyses of the ^90^Y PRRT subgroup

	Univariate analysis		Multivariate analysis	
Factors	HR and 95% CI	*P*	HR and 95% CI	*P*
**Age**				
<60				
≥60	1.647(1.130, 2.399)	0.009*	1.798(1.219, 2.653)	0.003*
**Gender**				
Male				
Female	1.400(0.968, 2.026)	0.074		
**^90^Y Therapy cycle**				
<4 cycles				
≥4 cycles	2.759(1.710, 4.451)	<0.001*	1.668(0.839, 3.318)	0.145
**Therapy group**				
^90^Y				
Combined	1.303(0.823, 2.065)	0.259		
**Follow-up time**	1.008(1.003, 1.012)	0.001*	1.006(1.001, 1.011)	0.012*
**^90^Y Cumulative dose**	1.090(1.043, 1.139)	<0.001*	1.051(0.988, 1.119)	0.115

## Abbreviations

PRRT: peptide receptor radionuclide therapy
NENs: neuroendocrine neoplasms
CTCAE: Common Terminology Criteria for Adverse Events
PFS: progression-free survival
OS: overall survival
EMA: European Medicines Agency
FDA: Food and Drug Administration
GEP-NETs: gastroenteropancreatic neuroendocrine tumors
SSTR2: somatostatin receptor subtype-2
EBRT: extrapolated from external-beam radiation therapy
PRCRT: peptide receptor chemo-radionuclide therapy
DOTA: 1,4,7,10-tetraazacyclododecane-N,N',N'',N'''-tetraacetic acid
GMP: Good Manufacturing Practice
HPLC: high-performance liquid chromatography
BBS: Bad Berka Score
GFR: glomerular filtration rate
TER: tubular extraction rate
