# Supplementary material for: Long-term Nephrotoxicity after PRRT: Myth or Reality
Source: Theranostics. 2024 Jan 1;14(2):451–9. doi: 10.7150/thno.92487 (PMC10758070; doi:10.7150/thno.92487)
Supplement: Supplementary file 1 — Supplementary figures and table. [file thnov14p0451s1.pdf]

Long-term Nephrotoxicity after PRRT: Myth or Reality

Supplemental Material

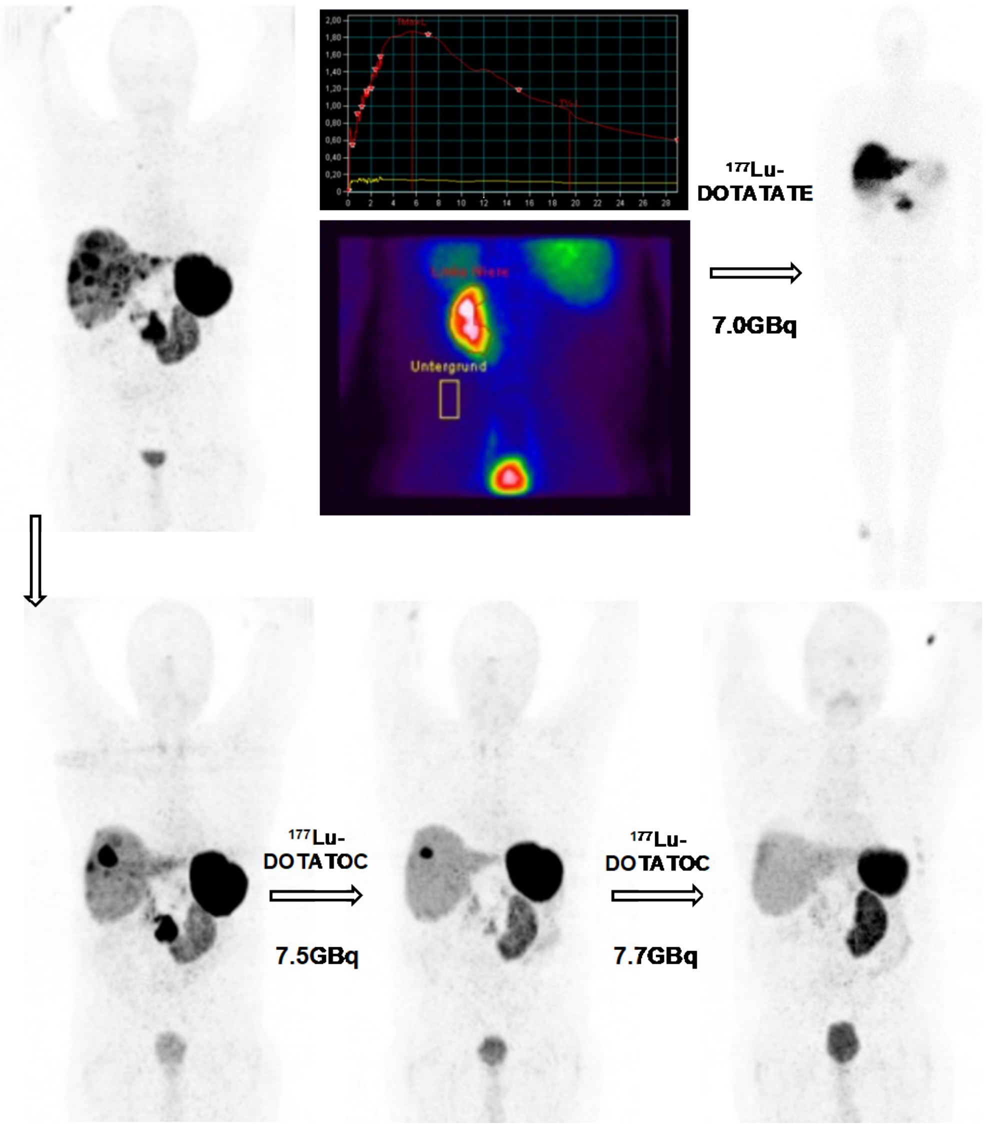

Figure. S1. This patient with single kidney (renal NEN had nephrectomy) and present with progressive live metastases. After reveived a total of 22.2 GBq and tumor lesions showed nearly complete remission as well as bulky lymph nedes located at the level of left kidney. Most importantly, his renal improved over time which was most probably due to tumor remission after PRRT.

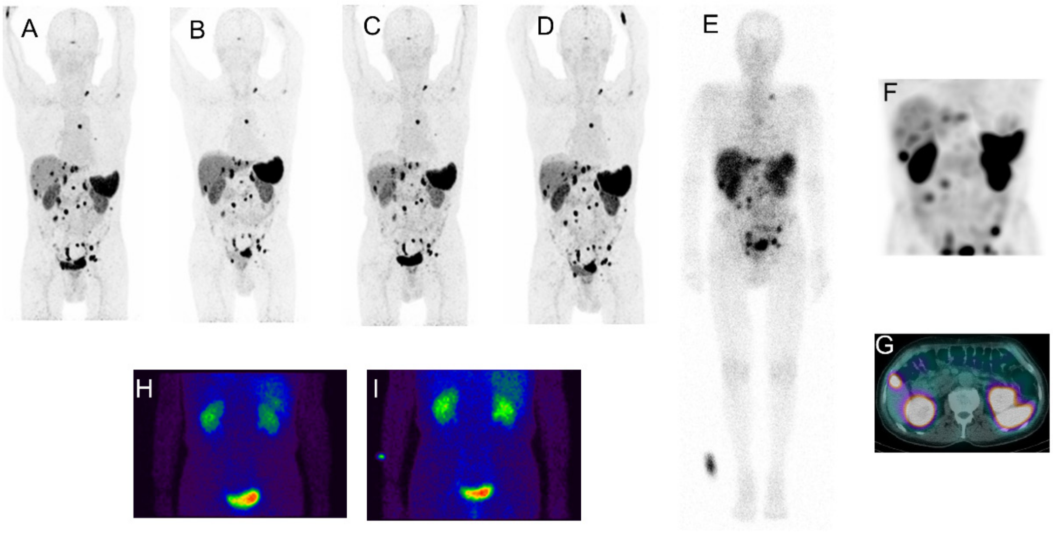

Figure. S2. This patient with pre-existing renal insufficiency (CTC G2) did not experience worsening after 3 cycles of ^{177}Lu-PRRT. A. ^{68}Ga-SSTR PET scan prior to PRRT treatment. B, C, D. Follow-up ^{68}Ga-SSTR PET scans after 1, 2, and 3 cycles of PRRT, respectively. E, F, G. ^{177}Lu-DOTATATE PET scans after 3 cycles of PRRT, showing the whole body, local, and cross-sectional views. H, I. TER imaging before (H) and after (I) PRRT treatment.

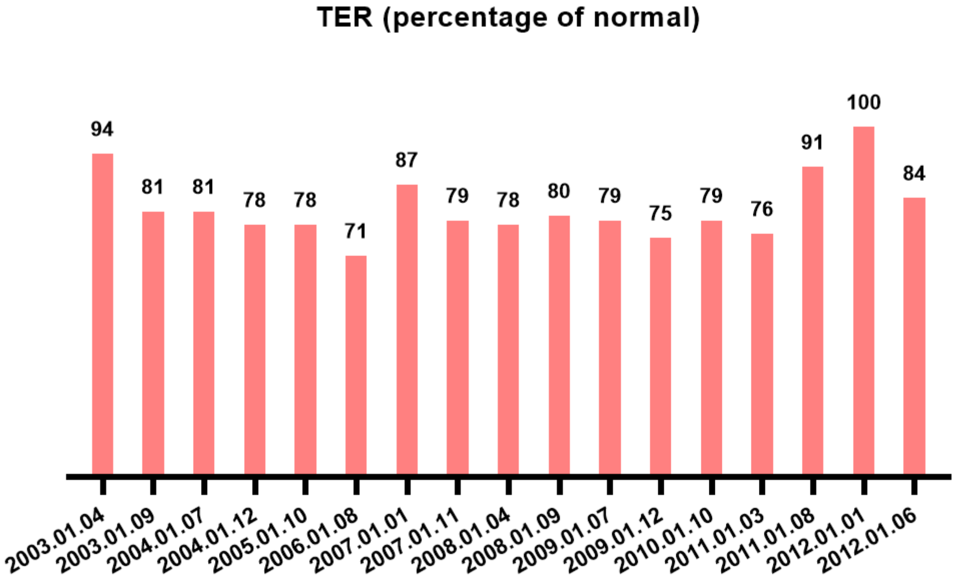

Figure. S3. Serial renal function assessment in a patient who underwent 10 PRRT cycles over a span of 9 years, using the measurement of the tubular extraction rate (TER) with ^{99m}Tc-MAG3.

Table. S1. Treatment cycles in 1361 patients

Radiopeptide	Number
Lu-177-DOTA-LM3	20
Lu-177-DOTA-NOC	16
Lu-177-DOTA-TATE	1476
Lu-177-DOTA-TOC	1736
Lu-177-HA-TATE	478
Lu-177-JR11	2
Y-90-DOTA-NOC	21
Y-90-DOTA-TATE	1056
Y-90-DOTA-TOC	562
Y-90-JR11	1
Y-90-OPS201	2
TANDEM	39
**In total**	5409
